# Cancer-associated fibroblast-derived fibulin-5 promotes epithelial–mesenchymal transition in diffuse-type gastric cancer via cAMP response element-binding protein pathway, showing poor prognosis

**DOI:** 10.1038/s12276-025-01447-8

**Published:** 2025-05-14

**Authors:** Jinju Choi, Yoonjin Kwak, Miree Park, Jeong Yeon Jo, Jun Hyuk Kang, Kook Myeong-Cherl, Hang-Rae Kim, Gwanghun Kim, Seong-Ho Kong, Do-Joong Park, Hye Seung Lee, Hyuk-Joon Lee, Jung Mogg Kim, Sang Gyun Kim, Han-Kwang Yang, Ji Kon Ryu, Soo-Jeong Cho

**Affiliations:** 1 Division of Gastroenterology, Department of Internal Medicine and Liver Research Institute, Seoul National University College of Medicine, Seoul, Republic of Korea; 2 Department of Gastroenterology, Seoul National University Bundang Hospital, Seongnam, Republic of Korea; 3 Department of Pathology, Seoul National University College of Medicine, Seoul, Republic of Korea; 4 Liver Research Institute, Seoul National University College of Medicine, Seoul, Republic of Korea; 5 Center for Gastric Cancer, National Cancer Center, Goyang, Republic of Korea; 6 Department of Biomedical Sciences, BK21 FOUR Biomedical Science Project, and Medical Research Center, Seoul National University College of Medicine, Seoul, Republic of Korea; 7 Department of Surgery and Cancer Research Institute, Seoul National University College of Medicine, Seoul, Republic of Korea; 8 Department of Microbiology, Hanyang University College of Medicine, Seoul, Republic of Korea

**Keywords:** Prognostic markers, Gastric cancer

## Abstract

Diffuse-type gastric cancer (DGC), characterized by poorly cohesive cells within fibrotic stroma, is associated with advanced disease and poor prognosis. Here, to identify distinct biomarkers for DGC compared with intestinal-type gastric cancer, we constructed a comprehensive large-scale signaling network using RNA-sequencing data from three genomic databases (The Cancer Genome Atlas, GSE62254 and GSE26253), developed a mathematical model and conducted simulation analyses. For validation, we used tissue microarray blocks of gastric cancers with immunohistochemical staining, single-cell RNA sequencing, primary cultures of cancer-associated fibroblasts (CAFs) and organoids, and a co-culture system involving CAFs and cancer cells. Signaling network analysis identified six differentially activated signaling components across the database, including *BIRC5*, *TTK*, *NEK2*, *FHL1*, *NR2F1* and *FBLN5*. Among the differentially activated signaling components, high tumoral expression of fibulin-5 protein encoded by *FBLN5* correlated with poor overall and disease-specific survival rates in patients with DGC, even after adjusting for the tumor, node, metastases (TNM) stage. Fibulin-5, derived from CAFs within DGC stroma, promoted organoid growth and epithelial–mesenchymal transition (EMT) in DGC cell lines via the cAMP response element-binding protein (CREB) pathway in a CAF co-culture system. *FBLN5* knockdown in CAFs reduced the aggressive phenotype of co-cultured DGC cells, while CREB inhibitors reversed EMT. Furthermore, levels of secreted FBLN5 in patient blood samples correlated with its expression in primary tumors. In summary, fibulin-5 secreted by CAFs and interacted with DGC cells promotes EMT and is clinically associated with poor patient outcomes. These findings suggest fibulin-5 as a potential prognostic marker and therapeutic target in patients with DGC.

## Fibulin-5 identified as key marker in diffuse gastric cancer

Gastric cancer is a major health concern worldwide, with two main types: intestinal and diffuse. Diffuse gastric cancer (DGC) is less common but more aggressive. Here researchers used advanced computer models to study the complex networks of genes and proteins in DGC. They focused on the protein fibulin-5 (FBLN5), which was found to be more active in DGC than in intestinal-type cancer. The study involved analyzing data from 1,139 patients and conducting experiments with cancer tissues and cells. They discovered that FBLN5, produced by cells called cancer-associated fibroblasts, helps cancer cells spread by promoting the epithelial–mesenchymal transition process. High levels of FBLN5 were linked to worse survival rates in patients with DGC. The authors concluded that FBLN5 could be a useful marker for predicting patient outcomes and a potential target for new treatments.

This summary was initially drafted using artificial intelligence, then revised and fact-checked by the author.

## Introduction

Gastric cancer is the fifth most common malignancy globally and ranks fourth in cancer-related mortality^1^. According to the Lauren classification, it is categorized into two types: intestinal and diffuse^2^. Each type has distinct clinical features, probably resulting from differences in genetic and molecular pathogenesis^3^. Given that genes and molecules operate within signaling networks, it is plausible that these two types differ not only in molecular and genetic expression, but also in their signaling network architecture. Cancer signaling networks are notably complex due to their extensive crosstalk and feedback loops^4^, requiring mathematical modeling and computer simulations to accurately assess the activity of signaling components^5^. While past research in gastric cancer has sought to identify key genes, proteins or pathways involved in the pathogenesis^6–8^, the methods have been static and unable to capture the dynamic nature of these networks.

Diffuse-type gastric cancer (DGC), although less prevalent, has poorer prognosis than the intestinal type. DGC consists of noncohesive scattered tumor cells surrounded by fibrotic stroma with abundant stromal cells including fibroblasts and immune cells in the tumor microenvironment^9^. It is associated with epithelial–mesenchymal transition (EMT), frequent metastasis and treatment resistance^10^. Recent trends show a stable-to-increasing incidence for this aggressive gastric cancer phenotype, which differs from the declining rates of intestinal-type cancers^11^. Thus, understanding the pathophysiology and investigating the treatment strategy for DGC have become critical areas of interest.

In this study, we constructed a large-scale signaling network, developed a mathematical model and conducted simulation analyses using RNA-sequencing data. This approach helped identify a biomarker that contributes to the aggressiveness and poor prognosis of DGC compared with intestinal type. We then investigated the function of this biomarker and explored its potential as a prognostic and therapeutic target.

## Materials and methods

### Normalized equation modeling and numerical simulation

A large-scale signal network was formulated as normalized differential equations and large-scale simulations were performed using parallel computers. See Supplementary Method 1 for details.

### Mapping of RNA-sequencing data to the signaling network

RNA-sequencing data from The Cancer Genome Atlas (TCGA), GSE62254 and GSE26253 datasets was preprocessed before the computer simulation as described in Supplementary Method 2.

### TMA and immunohistochemistry

Tissue microarray (TMA) blocks of 289 gastric cancer cases who underwent curative gastrectomy were constructed using a tissue array device (Beecher Instruments Inc.) from surgical specimens collected between May 2006 and March 2012 at Seoul National University Bundang Hospital (SNUBH). The study received approval from the SNUBH Institutional Review Board (IRB no. B-2401-879-103), and informed consent for tumor tissue research was obtained preoperatively from all patients. Data prospectively collected from the SNUBH gastric database were reviewed retrospectively for each patient. The end of the follow-up period was 5 March 2015, with a median follow-up duration of 60 months (range, 1–96 months).

Antibody information is listed in Supplementary Table 3. Quantitative analysis of immunohistochemical (IHC) staining in patient tissue samples was based on the histochemical score (*H* score), which was calculated by summing the staining intensity values multiplied by the percentage of cells showing that intensity; *H* score = (0 × *P*_0_) + (1 × *P*_1_) + (2 × *P*_2_) + (3 × *P*_3_). *P*_*x*_ refers to the percentage of cells at each intensity level. An intensity value of 0 means no staining, 1 indicates weak staining, 2 denotes moderate staining and 3 signifies strong staining.

### GSEA

Public datasets of gastric cancer samples (*n* = 415) were collected from TCGA and subjected to gene set enrichment analysis (GSEA). Genes were considered expressed if their normalized read counts across all samples exceeded 10. Based on *FBLN5* expression levels, the top and bottom 10% of the samples were classified as the *FBLN5*^high^ and *FBLN5*^low^ (*n* = 42 each), respectively. Differently expressed genes were defined by a fold change greater than 1.5 and a *P* value less than 0.05 between the two groups. Genes were then ranked and GSEA was performed using the GSEA tool (version 4.1.0).

Gene sets were curated from the Human RT² Profiler PCR array (SABiosciences) and the Kyoto Encyclopedia of Genes and Genomes. A statistically significant gene set was defined by normalized enrichment score (NES) greater than 1.5, a *P* value less than 0.05 and a false discovery rate (FDR) less than 0.25. The mRNA expression levels for each gene were normalized using *Z* score transformation and visually presented in a heat map.

### Single-cell RNA-sequencing

Fresh cancer tissue from patients with DGC was collected via forcep biopsy during endoscopic examinations and the process was approved by the Seoul National University Hospital (SNUH) IRB (IRB no. 2003-127-1110). The tissue was dissociated into single-cell suspensions using the tumor dissociation kit (Miltenyi Biotec, 130-095-929), following the manufacturer’s protocols. Data analysis was performed using the cell ranger program from 10X Genomics and Seurat v2.0. The adjusted *P* value from the Wilcoxon test comparing fibroblast_11 with all other cell clusters was 2.8 × 10^−304^.

### Patient-derived fibroblast isolation and culture

Gastric cancer and paired normal tissue samples from distant resection margins were collected from patients with DGC who underwent surgery at SNUH. All patients provided written informed consent before surgery, and the procedure was approved by the IRB of SNUH (IRB no. 2006-052-1131). Fibroblasts were isolated from both gastric cancer tissues (cancer-associated fibroblasts (CAFs)) and paired normal tissues (normal gastric tissue-associated fibroblasts (NAFs)) using the outgrowth method as described in previous studies^12,13^. The isolated fibroblasts were maintained in complete media. Their phenotype was confirmed by staining with known fibroblast markers. All experiments were conducted with fibroblasts maintained under nine passages.

### Live-cell imaging

MKN-45 cells were stably transduced with copGFP lentiviral particles (Santa Cruz Biotechnology, sc-108084) following the manufacturer’s protocol. The GFP-expressing MKN-45 stable cell line was seeded on 96-well plate on a high-content live-cell imaging system (PerkinElmer, Operetta CLS) and cultured for 96 h with or without NAFs or CAFs at a 5:1 ratio. Digital phase contrast images were captured every 30 min using a 20× high numerical aperture objective.

### Patient-derived GCOs

Patient-derived gastric cancer organoids (GCOs) were established following the methods described in a previous study^14^, with the approval of the SNUH IRB (IRB no. 1901-166-1007). Organoid area was measured using the Mshot Digital Image Analysis System program.

### Statistical analysis

All the experimental assays were conducted at least in triplicate. Bar plots are represented with mean ± s.e.m., as indicated by the error bars. The Pearson’s correlation test was employed to evaluate the correlation coefficient and its significance between two variables. Categorical variables from clinical data were analyzed using chi-square or Fisher’s exact test. Continuous variables were expressed as mean ± s.d. and analyzed using the Student’s *t*-test. Survival curves were constructed via the Kaplan–Meier method and compared using the log-rank test. The Cox-proportional hazards regression model was used to determine the effect of FBLN5 expression on mortality, while controlling for confounding covariates. A *P* value <0.05 was considered statistically significant. See Supplementary Method 3 for more details.

## Results

### DASCs between two Lauren-type gastric cancers identified via signaling network analysis using the SIGNOR database

**Fig. 1:**
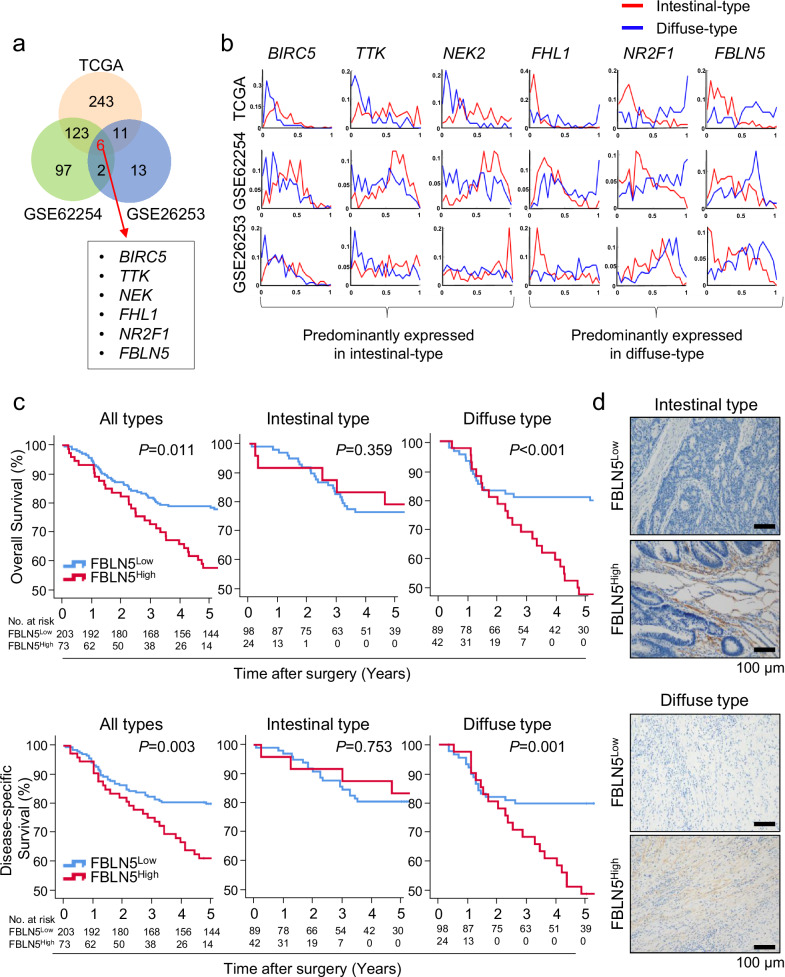
*FBLN5* was one of the DASC distinguishing between IGC and DGC, which was highly activated in DGC and has worse prognostic outcome. **a** A large-scale network analysis utilizing data from three genomic databases (TCGA, GSE62254 and GSE26253) identified six DASCs common to all three databases. **b** The activity distribution of the six DASCs among IGC and DGC patients. The *x* axis represents the scale of activity while the *y* axis is the estimated density in distribution. The red line represents the density distribution of the signaling component in IGCs while the blue line represents that of DGCs. **c** Kaplan–Meier curves for OS (top) and DSS (bottom) stratified by low versus high expression of FBLN5 in all cancers and each Lauren subtype. **d** Representative IHC staining images of FBLN^Low^ and FBLN^High^ cancer in each Lauren subtype (intestinal type, top and diffuse type, bottom). Scale bar, 100 µm.

To compare the dynamic characteristics of the two Lauren types, the SIGnaling Network Open Resource (SIGNOR) database (http://signor.urinoma2.it) was used to construct a large-scale signaling network. The network was then formulated as a mathematical model, followed by computer simulations using a parallel computing technique^15^ (refer to Methods for details and Supplementary Fig. 1a). The network, composed of 3557 nodes and 8509 links, demonstrated various signaling pathways and crosstalks (Supplementary Fig. 1b and Supplementary Table 1). Normalized equations were formulated for the different types of signal relationships included in the constructed network, creating a mathematical model (Supplementary Fig. 1c). Mathematical modeling facilitates the comparison of RNA-sequencing profiles across varying conditions by standardizing the state variables and parameters to values between 0 and 1, where 0 represents minimal activity and 1 represents maximal activity. Computer simulations of RNA-sequencing profiles from three large-scale patient databases (TCGA, GSE62254 and GSE26253) were performed using a parallel computing technique. A total of 1139 patients were included in the analysis: 407 from TCGA, 300 from GSE62254 and 432 from GSE26253. Given that simulations were conducted using one million initial conditions for each patient’s RNA-sequencing profile, one million activity profiles per signaling component were generated and represented as distributions (Supplementary Fig. 1d). Differentially activated signaling components (DASCs) between intestinal-type gastric cancer (IGC) and DGC were identified in each database. This analysis yielded 383 DASCs in TCGA, 228 in GSE62254 and 32 in GSE26253, with six DASCs common to all three databases (Fig. 1a and Supplementary Table 2). *BIRC5*, *TTK* and *NEK2* were predominantly activated in IGC, while *FHL1*, *NR2F1* and *FBLN5* were highly activated in DGCs (Fig. 1b). Notably, graph patterns across the six DASCs were similar in all three datasets.

### Elevated FBLN5 expression correlates with poor survival outcomes in DGCs within the validation cohort

To clinically validate the signaling network analysis described above, we investigated the protein expression levels of six previously identified DASC markers in a cohort of 298 patients with gastric cancer. TMAs comprising tissues from these patients were subjected to IHC staining for the six markers, and the expression levels of each marker were compared according to the two Lauren classifications. (Fig. 1d, Table 1 and Supplementary Fig. 2a–e). Among the six markers, FBLN5 and TTK showed statistically significant difference of expression patterns between IGC and DGC, consistent with the signaling network analysis. High FBLN5 expression was predominant in DGC (IGC versus DGC: 19.7% versus 32.1%, *P* = 0.031), while high TTK expression was more common in IGC than DGC (IGC versus DGC: 15.6% versus 3.1%, *P* = 0.0001) (Table 1).

**Table 1:** Expression levels of six proteins encoded by DASC genes inferred from signaling network analyses according to Lauren types.

		Intestinal (*n* = 122) *n* (%)	Diffuse (*n* = 131) *n* (%)	*P* value
BIRC5	high	112 (91.8%)	117 (89.3%)	0.528
	Low	10 (8.2%)	14 (10.7%)	
TTK	high	19 (15.6%)	4 (3.1%)	0.0001
	Low	103 (84.4%)	127 (96.9%)	
FHL1	high	30 (24.4%)	10 (7.6%)	<0.001
	Low	93 (75.6%)	121 (92.4%)	
NEK2	high	8 (6.6%)	5 (3.8%)	0.399
	Low	114 (93.4%)	126 (96.2%)	
NR2F1	high	61 (50.0%)	59 (45.0%)	0.452
	Low	61 (50.0%)	72 (55.0%)	
FBLN5	high	24 (19.7%)	42 (32.1%)	0.031
	Low	98 (80.3%)	89 (67.9%)	

To explore the clinical significance of these markers, both overall survival (OS) and disease-specific survival (DSS) were compared between patients with high or low expression of each marker. Survival analyses were conducted in three distinct groups: all Lauren-type gastric cancers, IGC and DGC patient groups. (Fig. 1c and Supplementary Figs. 2a–e and 3a–e). For FBLN5, cancer cases were categorized into FBLN5^Low^ and FBLN5^High^ groups based on the intensity of IHC staining in TMA tissues, and survival analyses were performed accordingly. FBLN5^High^ group showed poorer OS and DSS in the all-type and DGC patient cohorts, not among patients with IGC (Fig. 1c). We compared baseline characteristics between the FBLN5^Low^ and FBLN5^High^ groups (Table 2). The FBLN5^High^ group tended to include younger patients and showed a higher prevalence of cancers located in the upper and entire stomach, Lauren diffuse-type, greater tumor size and advanced tumor, node, metastases (TNM) tumor stages compared with the FBLN5^Low^ group.

**Table 2:** Comparison of baseline clinicopathologic characteristics between gastric cancer patient groups based on FBLN5 expression levels.

	FBLN5 low (*n* = 203)	FBLN5 high (*n* = 73)	*P* value
Age, years	61.39 ± 11.4	56.40 ± 11.3	0.001
Sex, *n* (%)			1.000
Male	127 (62.6%)	46 (63.0%)	
Female	76 (37.4%)	27 (37.0%)	
Location, *n* (%)			0.001
Lower third	102 (50.2%)	30 (41.1%)	
Middle third	74 (36.5%)	21 (28.8%)	
Upper third	26 (12.8%)	16 (21.9%)	
Entire	1 (0.5%)	6 (8.2%)	
Gross type, *n* (%)			0.127
Flat	16 (8.0%)	11 (15.7%)	
Protruded	34 (17.1%)	8 (11.4%)	
Depressed	149 (74.9%)	51 (72.9%)	
WHO classification, *n* (%)			0.103
TAWD	27 (13.3%)	9 (12.3%)	
TAMD	74 (36.4%)	15 (20.6%)	
TAPD	69 (34.0%)	36 (49.3%)	
SRC	27 (13.3%)	11 (15.1%)	
MAC	6 (3.0%)	2 (2.7%)	
Lauren type, *n* (%)			0.028
Intestinal	98 (48.3%)	24 (32.9%)	
Diffuse	105 (51.7%)	49 (67.1%)	
Tumor size (cm)	4.79 ± 2.64	6.24 ± 4.43	0.010
TNM stage, *n* (%)			0.006
Stage1	103 (50.7%)	28 (38.4%)	
Stage2	37 (18.2%)	8 (11.0%)	
Stage3	57 (28.1%)	29 (39.7%)	
Stage4	6 (3.0%)	8 (11.0%)	
Pathologic T stage, *n* (%)			0.010
pT1	101 (49.7%)	25 (34.2%)	
pT2	25 (12.3%)	5 (6.9%)	
pT3	37 (18.3%)	14 (19.2%)	
pT4a	36 (17.7%)	25 (34.2%)	
pT4b	4 (2.0%)	4 (5.5%)	
Pathologic N stage, *n* (%)			0.221
pN0	101 (49.8%)	30 (41.1%)	
pN1–3	102 (50.2%)	43 (58.9%)	

*MAC* mucinous adenocarcinoma, *SRC* signet-ring cell carcinoma, *TAMD* tubular adenocarcinoma, moderately differentiated, *TAPD* tubular adenocarcinoma, poorly differentiated, *TAWD* tubular adenocarcinoma, well differentiated, *WHO* World Health Organization.

A Cox-proportional hazard model was used to investigate the adjusted hazard ratio (HR) for overall and disease-specific mortality based on the level of FBLN5 protein expression. High FBLN5 expression was associated with an increased risk of overall and disease-specific mortality, after adjusting for confounding variables such as age, sex, tumor location, tumor size and TNM stage The adjusted HR for overall mortality was 1.515 (95% confidence interval (CI) 1.019–2.251, *P* = 0.040) and for disease-specific mortality was 1.673 (95% CI 1.013–2.762, *P* = 0.044) (Tables 3 and 4).

**Table 3:** A Cox-proportional hazards model for overall mortality in patients with gastric cancer (*n* = 276).

	Univariate			* Multivariate		
	HR	95% CI	*P* value	HR	95% CI	*P* value
Age	1.042	1.023–1.062	<0.001	1.053	1.033–1.073	<0.001
Sex						
Male	1 (reference)			1 (reference)		
Female	0.578	0.387–0.864	0.006	0.654	0.436–0.983	0.041
Location			<0.001			
Lower third	1 (reference)					
Middle third	0.621	0.398–0.968	0.035			
Upper third	1.505	0.936–2.420	0.092			
Entire	6.772	3.048–15.043	<0.001			
Size	1.224	1.168–1.283	<0.001	1.176	1.106–1.250	<0.001
Gross						
Flat	1 (reference)		0.287			
Protruded	0.567	0.279–1.150	0.116			
Depressed	0.235	0.404–1.249	0.235			
Lauren						
Intestinal type	1 (reference)		0.862			
Diffuse type	1.075	0.738–1.565	0.705			
Mixed type	1.182	0.601–2.325	0.628			
TNM						
Stage I	1 (reference)			1 (reference)		
≥Stage II	3.745	2.502–5.605	<0.001	2.867	1.821–4.516	<0.001
FBLN5						
Low	1 (reference)			1 (reference)		
High	1.626	1.107–2.390	0.013	1.515	1.019–2.251	0.040

^*^Adjusted by age, sex, tumor location, tumor size, TNM stage and FBLN5 expression.

**Table 4:** A Cox-proportional hazards model for disease-specific mortality in gastric cancer patients (*n* = 276).

	Univariate			* Multivariate		
	HR	95% CI	*P* value	HR	95% CI	*P* value
Age	1.007	0.986–1.028	0.531	1.026	1.004–1.049	0.021
Sex						
Male	1 (reference)			1 (reference)		
Female	0.629	0.375–1.057	0.080	0.590	0.344–1.012	0.055
Location			<0.001			
Lower third	1 (reference)					
Middle third	0.491	0.259–0.930	0.029			
Upper third	1.698	0.949–3.041	0.075			
Entire	6.839	2.838–16.482	<0.001			
Gross			<0.001			
Flat	1 (reference)		0.037			
Protruded	0.310	0.120–0.801	0.016			
Depressed	0.535	0.278–1.028	0.060			
Size	1.251	1.190–1.316	<0.001	1.183	1.105–1.266	<0.001
Lauren						
Intestinal type	1 (reference)		0.041			
Diffuse type	1.763	1.053–2.951	0.031			
Mixed type	2.276	1.018–5.089	0.045			
TNM						
Stage I	1 (reference)			1 (reference)		
≥Stage II	28.200	8.864–89.714	<0.001	17.668	5.437–57.418	<0.001
FBLN5						
Low	1 (reference)			1 (reference)		
High	2.021	1.253–3.262	0.004	1.673	1.013–2.762	0.044

^*^Adjusted by age, sex, tumor location, gross type, Lauren type, tumor size, TNM stage and FBLN5 expression.

Other markers that demonstrated statistical significance in survival analyses were as follows. High TTK expression was associated with OS specifically in IGC cohort (*P* = 0.035) (Supplementary Fig. 2b) and low NR2F1 expression was related to worse OS and DSS in all-type and IGC groups (*P* = 0.034 for OS in all types, *P* = 0.001 for DSS in all types, *P* = 0.013 for OS in IGC and *P* = 0.002 for DSS in IGC) (Supplementary Figs. 2e and 3e). We then chose to focus on FBLN5 as a marker due to its consistent results between signaling network analysis and clinical validation.

### FBLN5 originates from CAFs abundant in the tumor stroma

**Fig. 2:**
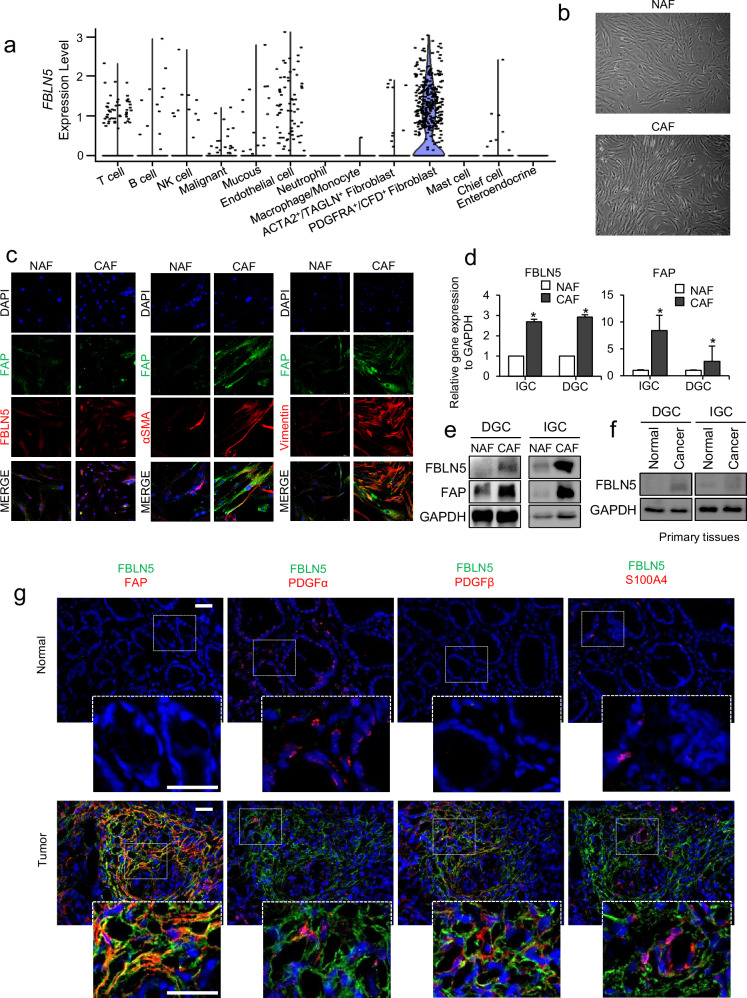
Fibulin-5 (FBLN5) is derived from CAFs in cancer stroma. **a** Single-cell RNA-sequencing data using four tissues from patients with DGC to investigate the origin of *FBLN5*. The dot plot shows the expression level of *FBLN5* across 13 identified cell clusters, highlighting its predominant derivation from fibroblasts. **b** Microscopic images of isolated fibroblasts obtained by the outgrowth method from paired normal (top) and cancer (bottom) tissue of actual patients with DGC. **c** Verification of PD-NAFs and PD-CAFs is conducted through immunofluorescent staining for CAF markers and FBLN5. **d** The relative mRNA expression of *FBLN5* and *FAP* is compared between PD-NAFs and PD-CAFs by RT–qPCR. *P < 0.05. **e** PD-NAFs and PD-CAFs, isolated from different Lauren DGC and IGC, are identified based on FAP and fibulin-5 expression levels through western blot analysis. **f** Fibulin-5 expression is compared in paired normal and primary tumor tissues from patients with DGC and IGC. **g** Immunofluorescent staining for CAF markers and fibulin-5 in paired normal and cancer tissues, accompanied by merged images showing the overlap of fibuilin-5 expression sites with CAF marker expression. DAPI (blue), FBLN5 (green) and CAF markers (FAP, PDGFα, PDGFβ and S100A4; red).

In the IHC staining of TMA blocks shown above, FBLN5 was predominantly localized in the stroma rather than in cancer cells (Fig. 1d). To explore the cellular origin of FBLN5 expression, we analyzed single-cell RNA-sequencing data from samples from four patients with DGC, given that the staining pattern of FBLN5 IHC confirmed that the protein was expressed in the stroma. Among the 13 identified cell clusters, the PDGFRA^+^/CFD^+^ fibroblast cluster, referred to as the CAF cluster, exhibited predominant *FBLN5* expression (Fig. 2a).

Fibroblasts were isolated from the paired normal and cancerous tissues of patients with gastric cancer using the outgrowth method and were named as patient-derived normal gastric tissue-associated fibroblasts (PD-NAFs) and patient-derived cancer-associated fibroblasts (PD-CAFs), respectively. The isolated cells exhibited a spindle shape under light microscopy (Fig. 2b). Immunofluorescence (IF) staining for CAF markers (fibroblast activation protein (FAP), α-SMA and vimentin) and FBLN5 confirmed the cell identity (Fig. 2c). The expression levels of FBLN5 and the active CAF marker FAP were higher in PD-CAFs compared with in PD-NAFs, as demonstrated by RT–PCR and western blot analyses. (Fig. 2d,e). In primary tissues, FBLN5 expression was higher in tumor samples compared with normal tissue, as shown by western blot analysis (Fig. 2f). IF staining of paired normal and cancer tissue samples also revealed high FBLN5 expression in cancer tissues, whereas expression was minimal in normal tissue. Furthermore, the merged IF images confirmed the origin of FBLN5 by demonstrating co-localization of CAF markers, including FAP, PDGFα, PDGFβ, S100A4 and FBLN5 (Fig. 2g).

### Fibulin-5 promotes EMT in DGC cells

**Fig. 3:**
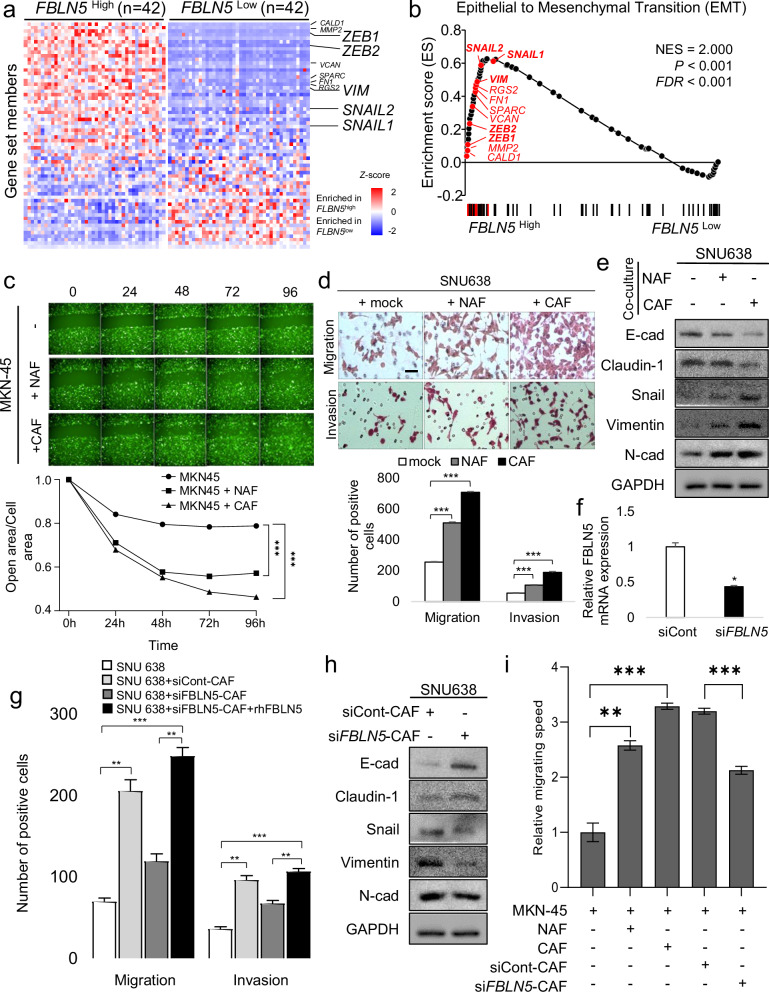
Fibulin-5 (FBLN5) promotes EMT of DGC. **a** A heat map generated from GSEA utilizing TCGA data illustrates the different gene distribution between *FBLN5*^Low^ and *FBLN5*^High^ patient cohorts. The mRNA expression levels, normalized via *Z* score transformation, indicate an enrichment of the EMT gene set in the *FBLN*^High^ group relative to the *FBLN5*^Low^ group. **b** Enrichment score plots (black curve) from GSEA for ranked genes within the EMT gene set reveal the NES, nominal *P* value and FDR. **c** Microscopic images of wound healing assays and corresponding line graphs depicting the relative open wound area over the cell area ratio at consecutive time points (hour) during live-cell imaging illustrate differences in wound healing between GFP-expressing MKN-45 stable cell line monoculture and co-cultures with PD-NAFs or PD-CAFs. ***P < 0.001. **d** A comparison of invasion and migration capabilities of the DGC cell line (SNU638) across cancer monoculture systems and co-culture systems with PD-NAFs and PD-CAFs represented through bar graphs. Scale bar, 50 µm. ***P < 0.001. **e** Western blot analysis of EMT markers in SNU638 cells under monoculture conditions and co-culture conditions with PD-NAFs or PD-CAFs. **f** Knockdown of *FBLN5* using si*FBLN5* in PD-CAFs co-culture systems compared with vehicle-treated PD-CAFs. *P < 0.02. **g** Invasion and migration assays of SNU638 cells in PD-CAF co-culture systems with or without si*FBLN5* treatment reveal significant decrease of these capabilities on knockdown of *FBLN5*. Restoration of FBLN5 using rhFBLN5 in co-culture system with si*FBLN5*-CAF restores migration and invasion of cancer cells, which were decreased after the knockdown of *FBLN5*. **P < 0.01 and ***P < 0.001. **h** A reduction in EMT marker expression in DGC cells following *FBLN5* knockdown in co-cultured CAFs compared with siControl (siCont) treatment in PD-CAFs is demonstrated. **i** Analysis of MKN-45 cell migration speed using the Operetta live-cell imaging system. Migration speed of GFP-expressing MKN-45 cells is compared under the conditions with the presence of patient-derived, NAFs, CAFs, siControl-treated CAFs and si*FBLN5-*treated CAFs. CAF-derived FBLN5 facilitates the migration of cancer cells, which was suppressed by si*FBLN5* treatment. The bars represent average speed of MKN-45 (µm/s) and s.d. **P < 0.01 and ***P < 0.001.

To identify differentially expressed genes associated with FBLN5 function in gastric cancer, GSEA was performed on the TCGA dataset (*n* = 415). Patients were classified into *FBLN5*^High^ (top 10%, *n* = 42) and *FBLN5*^Low^ groups (bottom 10%, *n* = 42) on the basis of their *FBLN5* expression levels (Fig. 3a). GSEA plots indicated significant enrichment of EMT-related genes in the *FBLN5*^High^ group compared with the *FBLN5*^Low^ group (NES of 2.0, *P* < 0.001, FDR of 0.001; Fig. 3b). To investigate the effect of CAF-derived fibulin-5 on EMT in DGC, co-culture systems were employed involving prescreened DGC cell lines and primary PD-CAFs (Supplementary Fig. 4a,b). Among DGC cell lines, MKN-45, SNU638 and SNU668 cell lines exhibiting low FBLN5 expression, were selected for further analysis. A wound scratch assay and a cell viability assay using a live-cell imaging system showed an enhanced rate of open wound closure and increased viability in the MKN-45 cell line when co-cultured with PD-CAFs or PD-NAFs (Fig. 3c and Supplementary Fig. 4c). The migration and invasion capabilities of the DGC cell line (SNU 638 and MKN-45) increased across cancer monoculture systems and co-culture systems with PD-NAFs and PD-CAFs (Fig. 3d and Supplementary Fig. 4d). When DGC cells (SNU 638 and MKN-45) were co-cultured with PD-NAFs and PD-CAFs, there was a decrease in the expression of epithelial markers E-cadherin and claudin-1, and alongside an increase in the expression of mesenchymal markers snail, vimentin and N-cadherin, referent to the monoculture system (Fig. 3e and Supplementary Fig. 4e). To further investigate the role of FBLN5, its expression was silenced in PD-CAFs using si*FBLN5* (Fig. 3f). Invasion and migration assays of SNU638 and MKN-45 cells in PD-CAF co-culture systems with or without si*FBLN5* treatment reveal a significant decrease of these capabilities on knockdown of *FBLN5* (Fig. 3g and Supplementary Fig. 4f,g). This reduction in migration and invasion was restored when FBLN5 was replenished by treating the co-culture system containing *FBLN5* knockdown CAFs with rhFBLN5 (Fig. 3g and Supplementary Fig. 4f,g). When *FBLN5* was silenced in CAF cells, the co-cultured DGC cells (SNU 638 and MKN-45) showed increased expression of E-cadherin and claudin-1. Conversely, there was a decrease in the expression of snail and vimentin (Fig. 3h and Supplementary Fig. 4h).

The migration speed of GFP-tagged MKN-45 cells, measured through live-cell imaging analysis, was higher in the CAF co-culture system. However, the relative velocity slowed down when *FBLN5* was silenced in co-cultured CAFs using siRNA (Fig. 3i). Supplementary materials include live-cell video imaging that support these finding (Supplementary Videos 1–5). The results indicated that CAF-derived FBLN5 significantly facilitates the migratory behavior of cancer cells.

### Secreted fibulin-5 is detectable in higher concentration in plasma samples from patients with DGC compared with normal samples

**Fig. 4:**
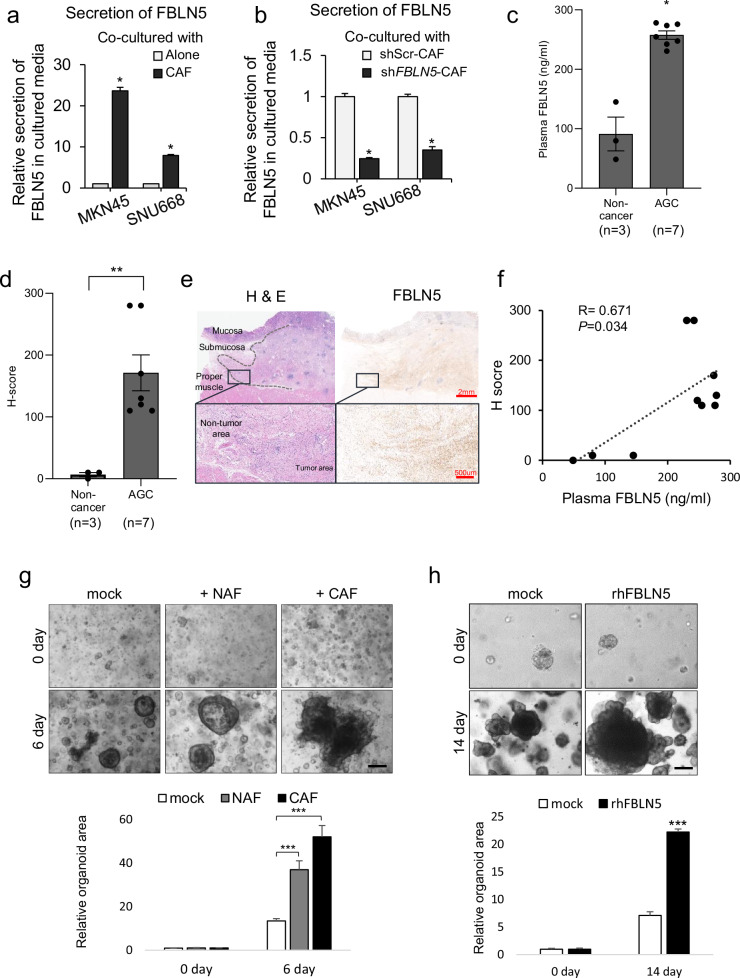
Secreted fibulin-5 (FBLN5) from CAFs is detectable in the blood of patients, paralleling its expression in primary tissue. **a** A comparison of FBLN5 concentration in culture media of monocultures from DGC cell lines (MKN-45 and SNU668) and co-cultures of these cancer cells with induced CAFs (iCAFs). **b** The relative concentration change of FBLN5 in culture media from the co-culture system following to the knockdown of *FBLN5* by transfection with sh*FBLN5* in iCAFs. **c** The concentrations of fibulin-5 (ng/ml), measured by ELISA in plasma samples, are compared between non-cancer subjects (*n* = 3) and patients with diffuse-type AGC (*n* = 7). *P < 0.05. d, The average *H* score, assessed from FBLN5 IHC staining, is compared between normal gastric tissues from non-cancer subjects (*n* = 3) and tumors from patients with AGC. **P < 0.01. **e** Representative paired H&E and IHC staining images for FBLN5 in AGC tissues display a gradient of expression from tumor to adjacent non-tumor tissues, indicative of local secretion and diffusion of FBLN. Scale bars, 2 mm and 500 µm (magnification ×10 and ×40). **f** Pearson’s correlation coefficient and scatter plot between the *H* score of gastric tissue and plasma FBLN5 concentration. **g** Microscopic examination of GCOs derived from an 83-year-old female patient with diffuse-type AGC shows different growth patterns among organoids cultured alone, with PD-NAFs and with PD-CAFs. Organoid areas were normalized to mean of day 0. The bar graph depicts the mean of individual organoid area per field for each group. Scale bar, 50 µm. ***P < 0.001. Scale bar, 50 µm. **h** Comparative microscopic images of GCOs treated with or without rhFBLN5 (10 ng/ml) illustrates an enhancement in organoid size with rhFBLN5 treatment, suggesting similar effect of CAF-derived FBLN5. Organoids area per field shown in bar graphs. ***P < 0.001. Scale bar, 50 µm.

To investigate how fibulin-5 from CAFs acts on DGC, culture media from the MKN-45 and CAF co-culture system was collected. The relative intensity of fibulin-5 expression was quantified by enzyme-linked immunosorbent assay (ELISA). Culture media withdrawn from the co-culture system showed significantly higher concentrations of fibulin-5 compared with the media from the cancer cell monoculture system (Fig. 4a). Conversely, media from the co-culture system containing *FBLN5*-silenced CAFs, with either MKN-45 or SNU 668, showed a marked reduction in fibulin-5 levels (Fig. 4b). These findings suggest the paracrine effect of fibulin-5 secreted from CAFs into the tumor microenvironment of DGC.

To assess the potential systemic effect of fibulin-5, blood samples from patients with DGC and non-cancer subjects for control were collected with informed consent, and the fibulin-5 concentration was measured using ELISA. The detailed clinical information is presented in Table 5. Plasma samples from patients with advanced DGC showed significantly higher fibulin-5 levels compared with controls (Fig. 4c). Similarly, IHC staining revealed a higher average *H* score (Methods) for FBLN5 in tumors from patients with advanced gastric cancer (AGC) (*n* = 7) compared with normal gastric tissues from non-cancer subjects (*n* = 3; *P* = 0.001) (Fig. 4d). Microscopic images for FBLN5 in AGC tissues showed a gradient of expression from tumor to adjacent non-tumor tissues (Fig. 4e). This pattern observed in paired hematoxylin and eosin (H&E) and IHC staining suggests localized secretion and diffusion of FBLN5 within the tumor microenvironment. Pearson’s correlation coefficient and scatter plot indicated that plasma FBLN5 concentration was positively correlated with the *H* score for FBLN5 in primary gastric tissue (*R* = 0.671, *P* = 0.034) (Fig. 4f), suggesting the potential utilization of plasma FBLN5 as a biomarker for cancer detection and tumor progression in gastric cancer.

**Table 5:** Baseline characteristics of ten individuals who were included in serum ELISA test and *H* score evaluation.

Patient no.	Age (years)	Sex	Diagnosis	Medical history	WHO histology	Lauren type	Cancer size	T stage	Metastatic LNs/examined LNs	N stage	M stage	Stage (AJCC 8th)	Treatment	Serum ELISA	*H* score
1	62	F	Gastric polyp	HTN	N/A	N/A	N/A	N/A	N/A	N/A	N/A	N/A	ESD	48.722	0
2	42	M	Duodenal polyp	DL	N/A	N/A	N/A	N/A	N/A	N/A	N/A	N/A	ESD	145.178	10
3	37	M	Gastric polyp	–	N/A	N/A	N/A	N/A	N/A	N/A	N/A	N/A	ESD	79.967	10
4	72	F	AGC	HTN	PCC	DT	8.0 × 7.0 × 1.1 cm	T2	16/48	N3a	M1	IV	STG	278.105	130
5	40	F	AGC	–	PCC	DT	3.0 × 2.3 × 0.6 cm	T2	10/51	N3a	M0	IIIA	STG	254.675	110
6	49	F	AGC	–	PCC	DT	14.7 × 6.3 × 1.0 cm	T3	8/52	N3a	Mo	IIIB	TG	230.608	280
7	53	M	AGC	HTN	PCC	DT	3.7 × 3.2 × 0.2 cm	T2	1/53	N1	M0	IIA	STG	275.885	110
8	55	M	AGC	–	PCC +TAMD	DT	6.9 × 3.0 × 0.2 cm	T2	0/47	N0	M0	IB	TG	247.576	120
9	58	F	AGC	–	PCC +TAMD	DT	9.5 × 7.0 × 1.1 cm	T4a	7/37	N3a	M0	IIIB	TG	273.732	170
10	51	M	AGC	–	PCC	DT	14.0 × 11.0 × 0.9 cm	T4a	34/70	N3b	M0	IIIC	TG	242.255	280

*DL* dyslipidemia, *DT* diffuse type, *ESD* endoscopic submucosal dissection, *HTN* hypertension, *LN* lymph node, *PCC* poorly cohesive carcinoma, *STG* subtotal gastrectomy, *TAMD* tubular adenocarcinoma, moderately differentiated, *TG* total gastrectomy.

Following the cell study, patient-derived GCOs were primarily cultured from an 83-year-old female patient with diffuse-type AGC. The organoid size was the largest in the CAF co-culture system, followed by the NAF co-culture condition, and the smallest in the GCO monoculture condition (Fig. 4g), which suggests that CAFs have an impact on promoting GCO growth similar to DGC cells. GCOs was treated with or without recombinant human (rh) FBLN5 (10 ng/ml), and the size of the organoid was serially compared during 14 days (Supplementary Fig. 5). GCOs showed an enhancement in organoid size with rhFBLN5 treatment (*P* < 0.001), suggesting similar effect of CAF-derived FBLN5 (Fig. 4h).

### Fibulin-5 promotes EMT in DGC through CREB pathway

We found out fibulin-5 was secreted from CAFs into the stroma, which accounts for the tumor microenvironment (Fig. 4) and this extracellular matrix protein could affect cancer cells resulting in increased EMT, and thus there may be an extracellular stimuli and intracellular signal transmission involving the process. We hypothesized that there may be a change of protein phosphorylation, which is the most common and pleiotropic modification in biology^16–18^.

**Fig. 5:**
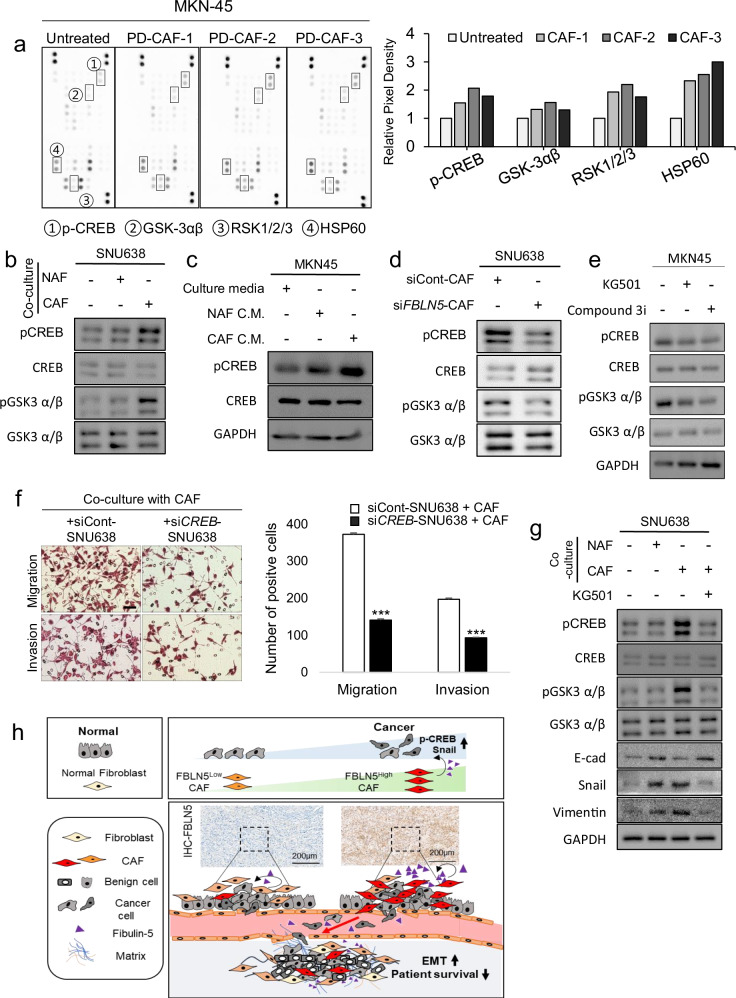
CAF-derived fibulin-5 (FBLN5) increases cancer aggressiveness through CREB signaling. **a** A screening phosphokinase array to investigate the kinase pathway related to the downstream signal of fibulin-5 on DGC cells, co-cultured with patient-derived CAFs from three patients with diffuse-type AGC. The analysis reveals upregulation in the pixel density of four kinases (pCREB, GSK-3αβ, RSK 1/2/3 and HSP60), as depicted in the accompanying bar graph. **b** Representative western blot images display the differential expression of signaling components between the monoculture system and CAF co-culture system. The expression levels of CREB-GSK components (pCREB Ser133 and pGSK-3αβ Ser21/9) in the DGC cell line are elevated in co-culture systems with either NAFs or CAFs compared with monocultures. The lowest expression is observed in monoculture, followed by higher levels in co-culture with NAFs, and the highest expression in co-cultures with CAFs. **c** Western blot images shows the expression of CREB signal components across different culture conditions, including control conditioned media (CM), NAF-cultured CM and CAF-cultured CM. The expression of phosphorylated CREB (pCREB Ser133) is enhanced in conditions containing CAF-cultured CM, followed by NAF-cultured CM. **d** Western blot analysis following the knockdown of *FBLN5* in CAFs reveals suppressed expression of CREB (pCREB Ser133) and GSK-3αβ (pGSK-3α Ser21/9) in the SNU 638 cells within the co-culture system. **e** The effects of CREB inhibitors (KG501 and Compound 3i (666-15)) on the MKN-45 cells are also assessed by western blot, showing decreased levels of phosphorylated CREB and phosphorylated GSK-3αβ upon treatment with these inhibitors. **f** Representative microscopic images from migration and invasion assays to evaluate the impact of CREB knockdown in DGC cells in a co-culture system with CAFs. The assays used SNU 638 cells and CAFs in co-culture systems with either a siControl or si*CREB*-treated DGC cell line. The number of migrating or invading cells are shown in the bar graphs. ***P < 0.001. Scale bar, 50 µm. **g** Western blot images of the expression of CREB-GSK signal components and EMT markers (E-cadherin, Snail and Vimentin) of SNU 638 cells co-cultured with either NAFs or CAFs. Treatment with CREB inhibitor KG501 reverses the effects induced by CAFs on SNU 638 cells. **h** An overview of the interplay between CAF-derived fibulin-5 and its influence on the aggressiveness of DGC through modulation of the CREB signaling pathway.

To investigate the mediators involved in the fibulin-5 signaling pathway, the phosphorylation profiles of protein kinases were compared between MKN-45 cells in monoculture system and those in a co-culture system with three different kinds of PD-CAFs isolated from patients with AGC. MKN-45 cells in the co-culture system showed an increase in relative phosphorylation levels of cAMP response element-binding protein (CREB), GSK-3αβ, RSK1/2/3 and HSP60 (Fig. 5a). Western blot assay showed the expression levels of CREB-GSK components (pCREB Ser133 and pGSK-3αβ Ser21/9) in DGC cell lines (SNU 638 and MKN-45) are elevated in co-culture systems with either NAFs or CAFs compared with monocultures. The lowest expression is observed in monoculture, followed by higher levels in co-culture with NAFs, and the highest expression in co-cultures with CAFs (Fig. 5b and Supplementary Fig. 6a). These results support our hypothesis that the CREB pathway in cancer cells may directly interact with CAF-derived fibulin-5 in DGC, suggesting that this signaling pathway plays a pivotal role in DGC progression. The expression of phosphorylated CREB (pCREB Ser133) was enhanced in conditions containing CAF-cultured media, followed by NAF-cultured media, compared with monocultured media (Fig. 5c). This finding suggests that CREB phosphorylation was also induced by secreted FBLN5. Furthermore, treating primary PD-CAFs with si*FBLN5* reversed the increased phosphorylation of pCREB (Ser133) and pGSK-3αβ (Ser21/9) in SNU 638 and MKN-45 cells (Fig. 5d and Supplementary Fig. 6b). Treating MKN-45 cells with CREB inhibitors, KG501 and Compound 3i (666-15), resulted in decreased levels of phosphorylated CREB (Ser133) and phosphorylated GSK-3αβ (Ser21/9) (Fig. 5e). Treatment with si*CREB* in SNU638 and MKN-45 cells reduced *CREB* mRNA expression (Supplementary Fig. 6c). The migration and invasion assays showed that *FBLN5* knockdown in CAFs decreased the migratory and invasive abilities of DGC cells (SNU 638 and MKN-45) (Fig. 5f and Supplementary Fig. 6d). The CREB inhibitor KG501 also reversed the effects induced by CAFs on SNU638 and MKN-45 cells, leading to decreased levels of phosphorylated CREB (Ser133), phosphorylated GSK-3αβ (Ser21/9), snail and vimentin, and increased E-cadherin expression (Fig. 5g and Supplementary Fig. 6e). Taken together, these findings indicate that interaction between CAF-derived fibulin-5 and its influence on the EMT and aggressiveness of DGC is mediated through the CREB signaling pathway within the tumor microenvironment, as shown in an overview (Fig. 5h).

## Discussion

In this study, six DASCs between DGC and IGC were identified by mathematical modeling and computer simulation analysis using large-scale RNA-sequencing data, which capture dynamic relationships among complex molecular networks. Among the six DASCs, *FBLN5* was shown to have a prognostic impact through TMA analysis of over 280 patients with cancer, incorporating detailed clinical information and survival data. The consistency between signaling analysis and TMA validation, as well as the association between biological data and clinical features, was a critical step of our study. Fibulin-5 expression levels strongly correlated with survival outcomes in patients with DGC, even after adjusting for confounding variables, including tumor stage. Furthermore, the presence of fibulin-5 in blood samples from patients with gastric cancer and its correlation with primary tumor tissue, suggests FBLN5 as a plasma biomarker for both treatment response and prognosis.

Fibulin-5 is a secreted extracellular matrix component known for its paracrine effect on cell adhesion^19–22^. The relationship between fibulin-5 and various cancers has been studied, but results vary based on primary tumor sites and clinical background^23^. In gastric cancer studies, high fibulin-5 expression correlates with more aggressive clinicopathologic features, such as poor differentiation, lymph node metastasis, advanced TNM stage and worse clinical outcome^24–27^. Our study provided more detailed clinical data and survival outcomes, confirming the negative prognostic impact of fibulin-5, consistent with previous findings. Experimentally, we demonstrated that fibulin-5 is secreted by CAFs, promoting EMT followed by poor patient survival (Fig. 5g). Fibulin-5 was even detectable in patient plasma, suggesting a systemic effect. In addition, plasma FBLN5 concentration positively correlated with FBLN5 protein expression of the primary tumor tissue, indicating that plasma fibulin-5 levels represent its expression in gastric tumors. This finding suggests that blood fibulin-5 could serve as a tumor marker in patients with DGC.

NAFs used in Figs. 2 and 3 are different from ‘normal fibroblasts’ isolated from normal gastric tissue. We took paired normal and cancer tissue samples from patients with AGC. Therefore, normal tissue was taken from adjacent normal-looking stomach surrounding tumors, and we assumed these NAFs as normal fibroblasts as a control. Different from our expectation, however, NAFs also showed FBLN5 expression and promoted proliferation, although the effect was less than that of CAFs, which was in line with previous studies^28–30^. We hypothesize that the reason NAFs also secrete fibulin-5 and promote cell migration and invasion is that normal fibroblasts may become activated in the adjacent normal tissue surrounding tumors by cytokines, or activated fibroblasts may be recruited from cancers. Further study will shed light on this interesting topic.

The probable pathway within cancer cells induced by secreted fibulin-5 is the CREB–GSK-3αβ pathway, based on results from the phosphokinase array and other supporting experiments in the present study. Elevated CREB expression is known to be related to cancer migration, metastasis and poor survival in many cancers, making this pathway a therapeutic target^31^. Knocking down *FBLN5* significantly reduced CREB activation, and CREB inhibitors replicated the effects of *FBLN5* inhibition, which showed a close correlation between the two factors. However, a limitation of this study is the inability to identify the exact mechanism by which fibulin-5 activates the CREB–GSK-3αβ pathway, ultimately resulting in cancer progression through EMT enhancement in DGC. Our study showed that fibulin-5 may regulate the CREB–GSK-3αβ pathway in the EMT process in DGC, suggesting that inhibiting fibulin-5 can be a treatment target of DGC. A hypothesis is that fibulin-5 binds to integrins on cancer cells and triggers the downstream CREB pathways^31,32^. Further experimental studies are needed to elucidate the precise mechanism involved. Another limitation is that clinical validation was conducted using cases from the Asian population. Nevertheless, FBLN5 was identified through genomic studies across various ethnicities and was consistently found in all three genomic databases. While further validation is necessary, FBLN5, as a biomarker, has the potential to be applicable to other ethnicities and races.

In conclusion, high FBLN5 protein expression is linked to worse OS and DSS in patients with DGC. In the fibrotic stroma of DGC, CAF-derived FBLN5 promotes EMT through the downstream CREB pathway and is secreted into bloodstream, where it correlates with expression in primary tumor tissue. Therefore, FBLN5 serves as both a prognostic biomarker and a potential therapeutic target.

## Supplementary information

Supplementary Information

Supplementary Table 1.

Supplementary Table 2.

Supplementary Video 1.

Supplementary Video 2.

Supplementary Video 3.

Supplementary Video 4.

Supplementary Video 5.
