# Supplementary material for: Cancer-associated fibroblast-derived fibulin-5 promotes epithelial–mesenchymal transition in diffuse-type gastric cancer via cAMP response element-binding protein pathway, showing poor prognosis
Source: Exp Mol Med. 2025 May 14;57(5):1106–19. doi: 10.1038/s12276-025-01447-8 (PMC12130531; doi:10.1038/s12276-025-01447-8)
Supplement: Supplementary file 1 — Supplementary Information [file 12276_2025_1447_MOESM1_ESM.pdf]

Supplementary Table 1. Detailed information of cancer signaling network. Refer to the excel file (i.e., Supplemental_Table_1.xlsx). The sheet "node" contains the information of the name, type, and UniprotKB identifier for entire 3,557 nodes. The sheet "link" contains the information of the nodes consisting the link and the type of the relationship for entire 8,509 links.

Supplementary Table 2. Detailed information of the six DASCs. Refer to the excel file (i.e., Supplemental_Table_2.xlsx).

Supplementary Table 3. Detailed information of primary antibodies for immunohistochemical staining.

FBLN5	1:50, Atlas, HPA000868
BIRC5	1:500, Abcam, ab76424
TTK	1:25, Thermo Fisher, 35-9100
NEK2	1:1000, AbnobaTM, H00004751-M01
FHL1	1:100, Atlas, HPA001040
NR2F1	1:400, Abcam, ab181137

Supplementary Figure Legends

Supplementary Fig. 1. Comprehensive analysis of a reconstructed large-scale signaling network for study. (a) Scheme for analysis of the large-scale cancer signaling network. Investigative process initiates with the construction of a substantial signaling network (Step 1). Subsequently, the network is modeled employing normalized equation modeling method. Numerical simulation are conducted utilizing 'ode15s' function in MATLAB (Step 2). The activity of signaling components is then compared between intestinal type and diffuse type tumors (Step 3). (b) Network composition. The network consists of 3,557 nodes interconnected by 8,509 links. Each dot represents the node and each line represents the link (red line for activation link and blue line for inhibition link). The network is reconstructed based on SIGNOR database (https://signor.uniroma2.it) (c) Mathematical model formulations: (i) Link formulation "X activates Y". (ii) Link formulation "X inhibits Y". (iii) Combined link formulation "X activates Y and Z inhibits Y". (d) Identification of differentially activated signaling components (DASCs): Simulation of the mathematical model using processed RNA sequencing data results in the distribution of activity of the signaling components. Since one million simulations were performed using one million different initial conditions, one million values for each signaling components finally come out and can be represented as a form of distribution. By comparison of the distributions, DASCs can be investigated.

Supplementary Fig. 2. Overall survival analysis and immunohistochemical (IHC) staining of tissue microarray (TMA) blocks in a clinical validation cohort of gastric cancer patients. Overall survival (OS) is compared across patient groups stratified according to the expression levels of five differentially activated signaling components (DASCs), identified through signaling network analysis. The OS differences are assessed based on the levels of DASC expression both in total patient cohort and within subgroups categorized by Lauren classification. Paired IHC staining of TMA blocks is shown in the right side of each OS graph illustrating representative patterns of low and high DASC expression in both intestinal- and diffuse-type gastric cancers. (a) BIRC5 (b) TTK (c) NEK2 (d) FHL1 (e) NR2F1. Scale bar 100μm.

Supplementary Fig. 3. Disease-specific survival analysis in a clinical validation cohort of gastric cancer patients. Disease-specific survival (DSS) was compared between patient groups categorized by the expression levels of five differentially activated signaling components (DASCs), as identified through signaling network analysis. The DSS was evaluated both in overall patient cohort and within subgroups stratified according to Lauren subtypes.

Supplementary Fig. 4. Co-culture system consisting of cancer cells and cancer-associated fibroblasts (CAFs). (a) The basal expression level of fibulin-5 (FBLN5) was assessed using western blot analysis across various gastric cancer cell lines prior to conducting *in vitro* cell studies. MKN45, SNU638, and SNU668 which are diffuse-type gastric cancer (DGC) cell lines exhibiting low FBLN5 expression, were selected for further investigation into the role of CAF-derived FBLN5. (b) Comprehensive figures of three different co-culture system comprised of diffuse gastric cancer cell line and CAF used in this study. a. Direct co-culture system of cancer cells and CAFs. b. Indirect co-culture system involving cancer cells and CAFs using trans-well method. c. Indirect system using conditioned media of co-cultured cancer cells and CAFs. (c) Cell viabilities are assessed using Operetta live cell imaging device in mono-culture system of MKN45 cells with GFP tagging and co-culture systems consisting of cancer cells paired with either patient-derived normal gastric tissue-associated fibroblasts (PD-NAFs) or PD-CAFs. Viable DGC cells co-cultured with NAFs shows a 1.11-fold increase relative to mono-culture system. Viability is enhanced by 1.57-fold compared to cancer cell mono-culture. ** $P<0.01$, *** $P<0.001$ (d) Comparison of invasion and migration capabilities of MKN45 cells across cancer mono-culture systems and co-culture systems with PD-NAFs and PD-CAFs is represented through bar graphs. The lowest cell number is observed in mono-culture, followed by higher level in co-culture with NAFs, and the highest number in co-cultures with CAFs. *** $P < 0.001$. Scale bar 50µm. (e) Western blot analysis of EMT markers in MKN45 cells under mono-culture conditions and co-culture conditions with PD-NAFs or PD-CAFs is depicted. (f) Representative images of invasion and migration assays of SNU638 cells in PD-CAF co-culture systems, with or without si*FBLN5* treatment, and with restoration of FBLN5 using rhFBLN5. Scale bar 50 µm. (g) Representative images of invasion and migration assays of MKN45 cells in PD-CAF

co-culture systems with or without si*FBLN5* treatment reveal significant decrease of these capabilities on knockdown of *FBLN5*. ******* *P* < 0.001. Scale bar 50 μm. (h) Reduction in EMT marker expression in MKN45 cells following *FBLN5* knockdown in co-cultured CAFs compared to siControl treatment in PD-CAFs is demonstrated.

Supplementary Fig. 5. Investigation for secreted fibulin-5 and the effect on diffuse-type gastric cancer organoids (GCOs). Representative microscopy of GCOs under the conditioned media with or without human recombinant FBLN5 (rhFBLN5). Scale bar 50 μm.

Supplementary Fig. 6. Experiments showing the possible relations between fibulin-5 and c-AMP response element binding protein (CREB). (a) Representative western blot images display the differential expression of signaling components between mono-culture system and CAF co-culture system. The expression levels of CREB-GSK components (pCREB Ser133 and pGSK-3αβ Ser21/9) in MKN45 cell line are shown in co-culture systems with either normal gastric tissue-associated fibroblasts (NAFs) or CAFs compared to mono-cultures. (b) Western blot analysis following the knockdown of *FBLN5* in CAFs reveals suppressed expression of CREB (pCREB Ser133) and GSK-3αβ (pGSK-3α Ser21/9) in the MKN45 cells within the co-culture system (c) A comparative analysis of *CREB* mRNA expression levels in SNU638 and MKN-45 cell lines following treatment either siCont or si*CREB*. * *P* < 0.05. (d) Representative microscopic images from migration and invasion assays to evaluate the impact of CREB knockdown in MKN45 cells in co-culture system with CAFs with either siControl or si*CREB*-treated cancer cell line. The number of migrating or invading cells shown in bar graphs. ******* *P* < 0.001. Scale bar 50 μm. (e) Western blot images of the expression of CREB-GSK signal components and EMT markers (E-cadherin, Snail, Vimentin) of MKN45 cells co-cultured with either NAFs or CAFs. Treatment with CREB inhibitor KG501 reverses the effects induced by CAFs on DGC cells.

Supplementary Figure 1

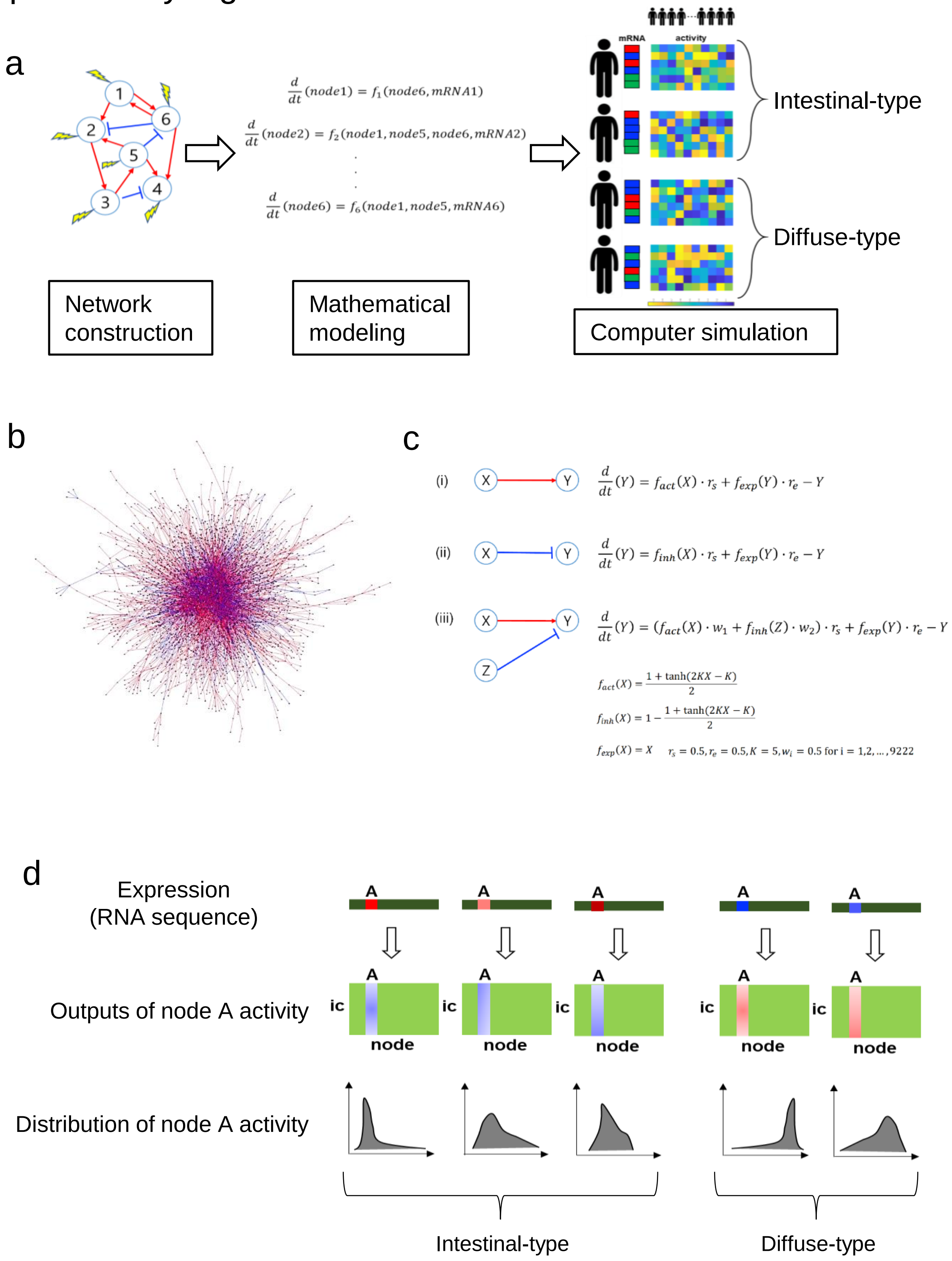

Supplementary Figure 2

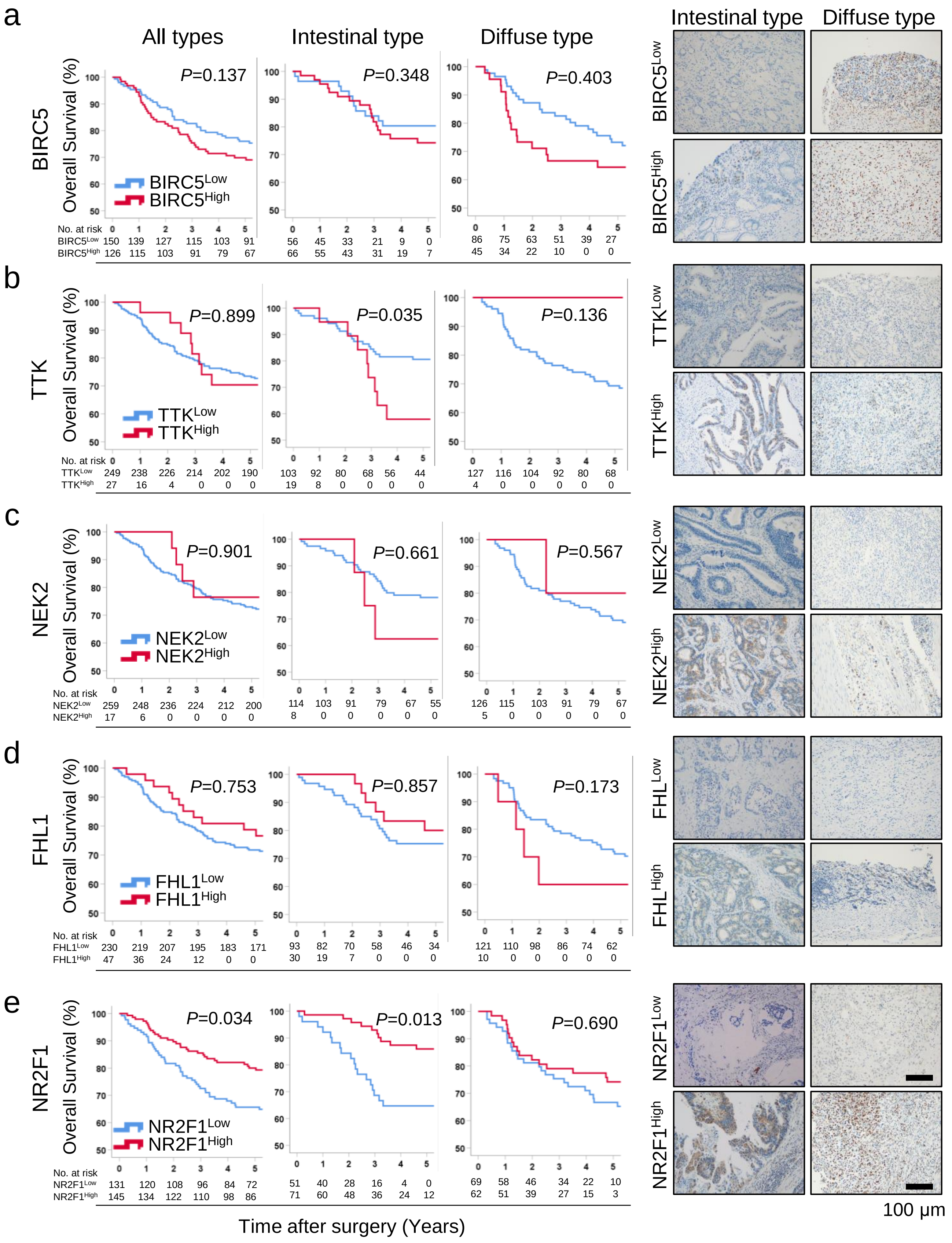

Supplementary Figure 3

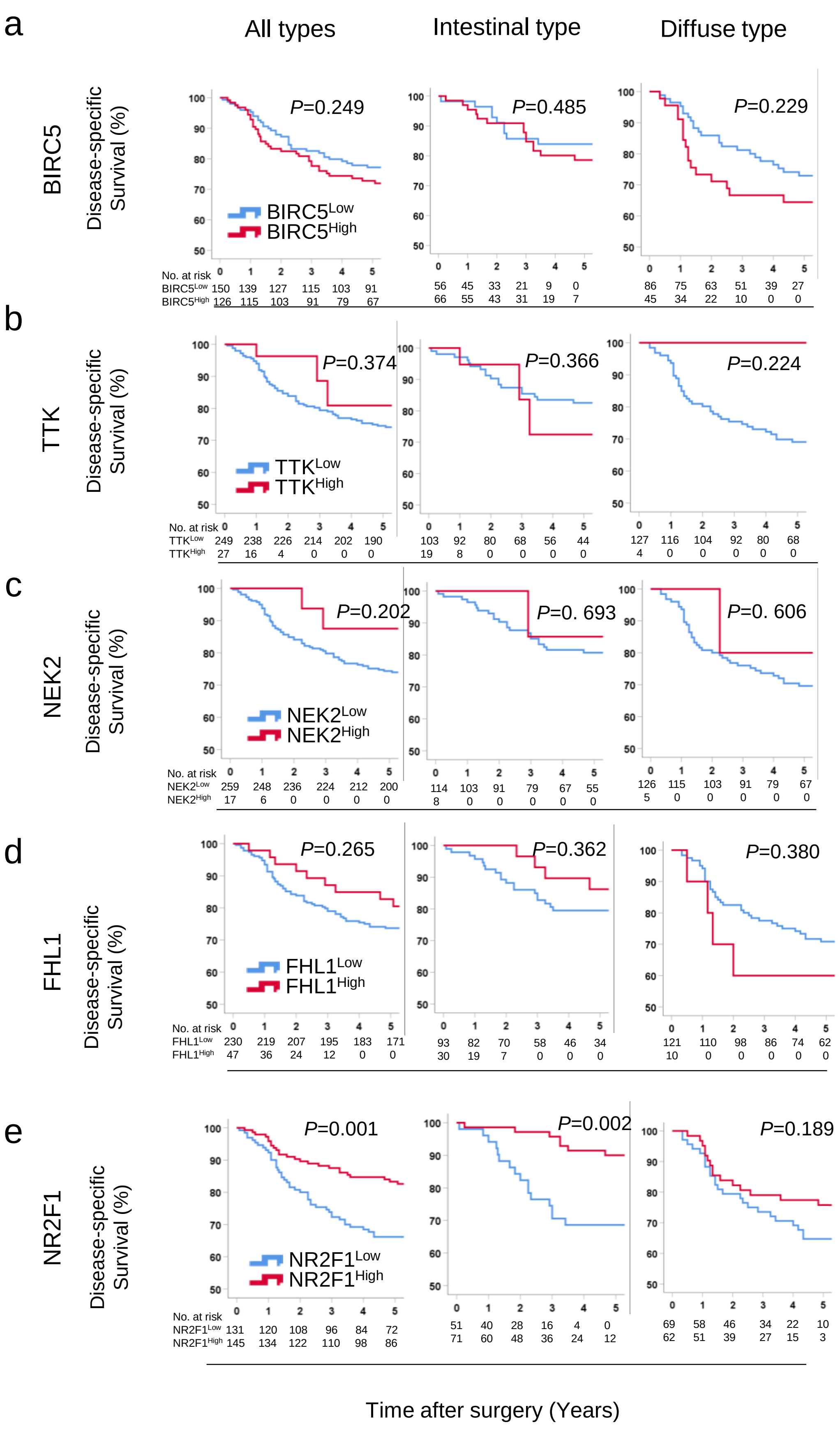

Supplementary Figure 4

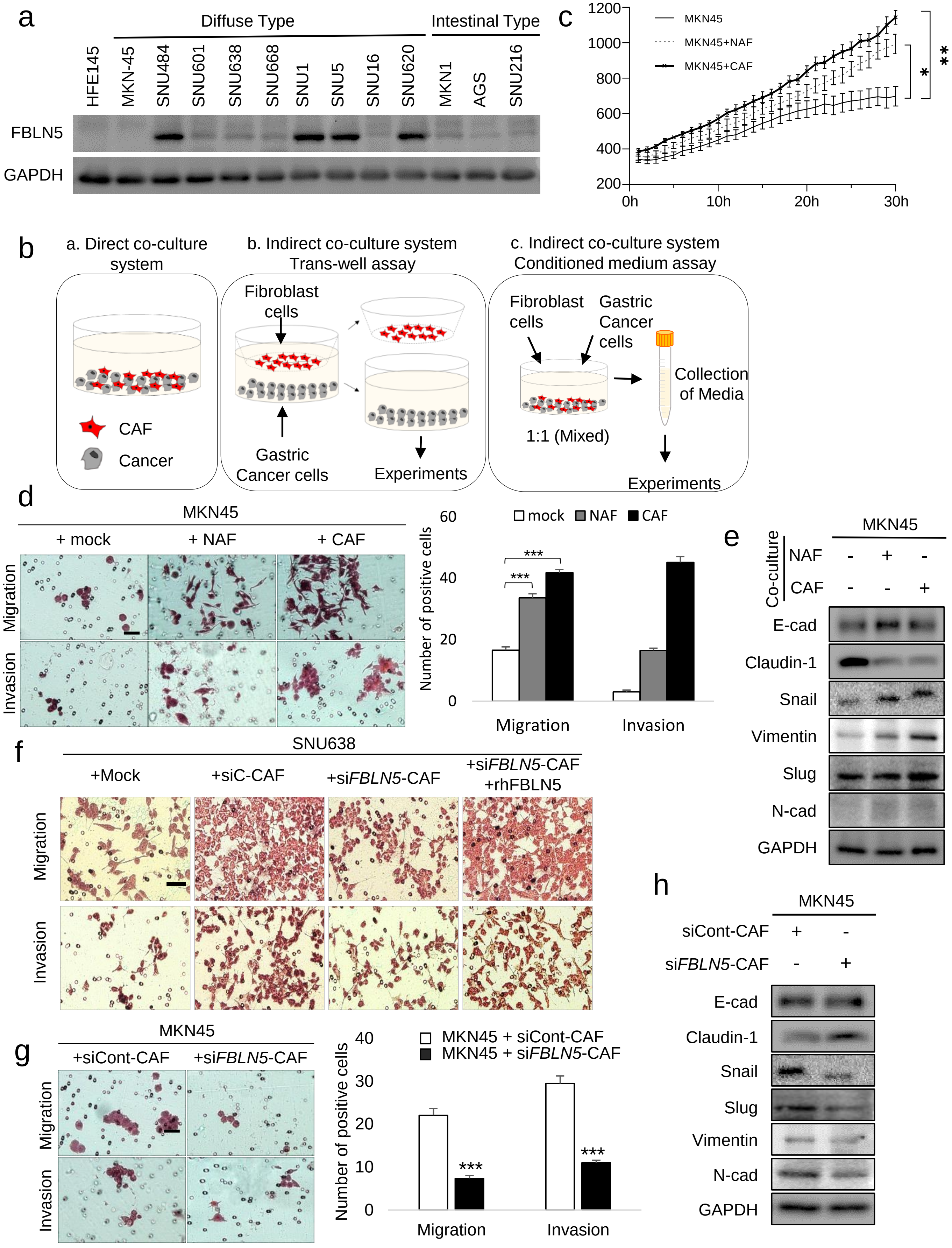

Supplementary Figure 5

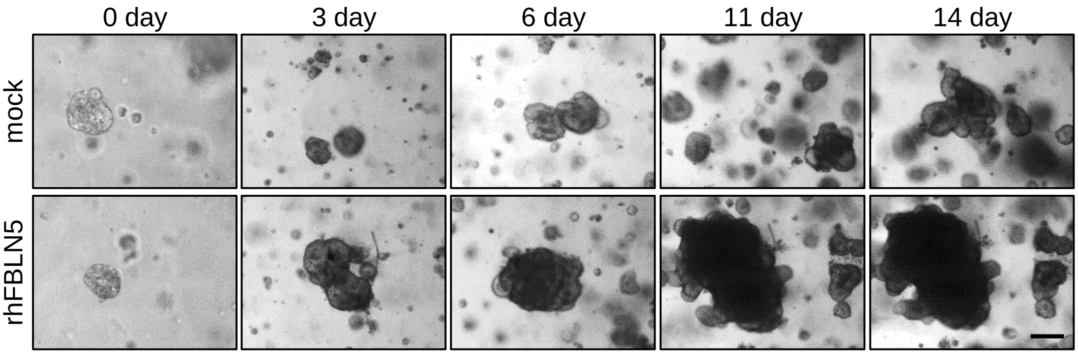

Supplementary Figure 6

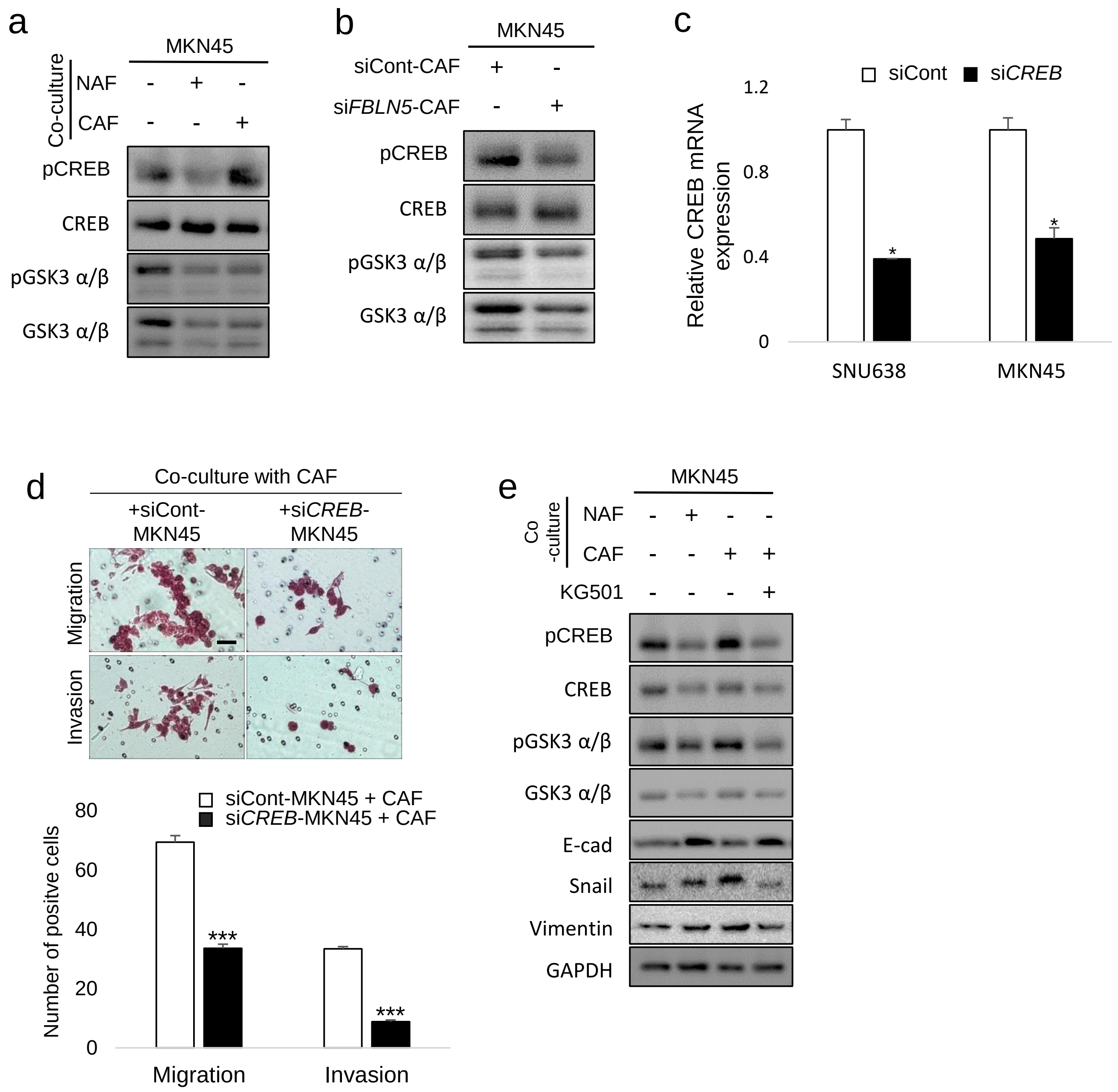

Supplementary Videos. Live cell video imaging. The migration speed of GFP-expressing MKN45 cells was measured using the Operetta live cell imaging system. The migration speed of individual GFP-expressing MKN45 cells was assessed and compared under various conditions. MKN45 cells under mono-culture (Supplementary Video 1) and MKN45 cells in the presence of patient-derived normal gastric tissue-associated fibroblasts (NAFs; Supplementary Video 2), CAFs (Supplementary Video 3), siControl-treated-CAFs (Supplementary Video 4), and si*FBLN5*-treated CAFs (Supplementary Video 5). Migratory cells were visualized using green fluorescence.

Supplementary method 1. Normalized equation modeling and numerical simulation

In a network model based on its topology, each component's dynamics are captured through a set of normalized differential equations. The activity of each node is constrained between 0 (minimum activity) and 1 (maximum activity). The configuration of these differential equations depends on the number of incoming links to a node and the nature of these links, whether activating or inhibiting. If node Y is regulated by another node X, the instantaneous rate of change in Y (dY/dt) is determined by the activity of X and the expression of Y (Supplementary Fig. S1C (i), (ii)). If node Y is regulated by two other nodes, X and Z, dY/dt is determined by the sum of the three influences: the individual influence of X and Z on Y, as well as the expression of Y itself (Supplementary Fig. S1C (iii)). The equation includes four parameters, rs, re, w1, w2: rs represents the combined influence of X and Z on Y and re represents the influence of expression of Y on its own activity; w1 represents the influence of X on Y and w2 represents the influence of Z on Y. To simplify, rs and re were set to 0.5, and weighting parameters were determined by 1 divided by the number of influencing nodes. In the case of two influencing nodes (X and Z), w1 and w2 are each set to 0.5. If Y is regulated by three nodes, w1, w2, and w3 would each be 0.33.

Then, numerical simulations for the differential equation system were executed using the ‘ode15s’ function in MATLAB until stabilization was achieved across a million random initial conditions. Given that the total number of simulations reached 1.1 billion, these large-scale simulations were carried out on a high-performance, 64-core computer using parallel computation.

Supplementary method 2. Mapping of RNA sequencing data to the signaling network

Information regarding causal relationship between various biological entities (such as mRNA, protein, phenotype, etc.) for a large signaling network was collected from SIGNOR database [1]. To minimize uncertainty within the network, the original database underwent a series of pre-processing steps: (i) indirect/unknown links removal: links representing indirect of unknown effects were removed. (ii) transcriptional regulation links removal: links representing transcriptional regulation were removed because the model operates with pre-defined set of expression levels. (iii) node type exclusion: nodes representing chemical, microRNA, fusion protein, phenotypes were removed. (iv) duplicated links removal. (v) incoherent links removal: links representing simultaneous activation and inhibition were removed as their exact regulatory nature was uncertain. (vi) self-link removal: links activating or inhibiting themselves were removed.

RNA sequencing data from The Cancer Genome Atlas (TCGA), GSE62254, and GSE26253 datasets were acquired in FPKM format using the R programming. To obtain RNA sequencing data from TCGA-STAD, R packages of TCGAWorkflowData, DT, TCGAbiolinks, httr, and jsonlite were used. The GEOquery, Biobase, and limma packages were used to obtain data from GSE62254 and GSE26253. The Ensemble gene identifier was converted into UniProtKB identifier by using ID mapping function provided by Uniprot. The assigned value of certain UniProtKB identifier was regarded as baseline gene expression of corresponding signaling component (www.uniprot.org). These values were mapped to the constructed signaling network, and the mapping rates (the fraction of nodes which have baseline expression values among all nodes belonging to the network) were 3131/3557, 3241/3557, and 2601/3557 in TCGA, GSE62254, and GSE26253, respectively. Unmapped nodes were assigned the average expressions value of all nodes within the network. The baseline expression values were normalized using z-score transformation and cumulative distribution functions, where the lowest value was set to 0 and the highest to 1. These normalized values were then incorporated as variables into the normalized equation model.

Supplementary method 3.

Cell lines and reagents

The DGC cell lines SNU1, SNU5, SNU16, SNU484, SNU601, SNU638, and MKN45 as well as the intestinal-type gastric cancer cell lines AGS, NCI-N87, MKN1, MKN74, SNU216, and SNU719, were obtained from the Korean Cell Line Bank (Seoul, Republic of Korea). The immortalized human normal gastric epithelial cell line, HFE145, was generously provided by Dr. Hassan Ashktorab and Duane T. Smoot (Howard University, Washington, DC), and was maintained in RPMI 1640 medium. All cancer cell lines were cultured in DMEM or RPMI, and were actively passaged for less than 3-6 months after acquisition, in accordance with United Kingdom Coordinating Committee on Cancer Research guidelines [2]. All culture media were supplemented with 10% FBS, 1% penicillin/streptomycin, and 2 mM L-glutamine.

Real-time PCR

RNA extraction and amplification was conducted as previously described [3]. All reactions were performed in triplicate. A comparative threshold cycle method (ΔCT) was used for comparison, with values expressed as 2-ΔΔCT. The sequences of the primers are as follows: *FAP* Sense: 5'-TGTGCATTGTCTTACGCCCT-3' and Antisense: 5'-CCGATCAGGTGATAAGCCGT-3'; *FBLN5* Sence: 5'-ACTCGACCCCCTACTCAGG-3' and Antisense: 5'-TGTCGCTATGGTTACTGCCA-3'; *CREB* Sence: 5'-ATTCACAGGAGTCAGTGGATAGT-3' R: 5'-CACCGTTACAGTGGTGATGG-3'.

Immunocytochemistry

Cells were seeded in 12-well plates, and after 24h, were fixed in 4% formaldehyde for 10 minutes, followed by washing and pre-blocking. The cells were then incubated with primary antibodies, anti-fibulin-5 antibody (Abcam, ab66339), anti-FAP antibody (Cell Signaling, #66562) to identify CAFs. Following the washing steps, cells were incubated with fluorescent-conjugated secondary antibody for 1 hour. For nuclear staining, DAPI (Vector, VECTASHIELD® Antifade Mounting Medium with DAPI, #H-1200) was applied. Images were captured using confocal microscopy (Leica STDE CW).

Protein extraction and western blot

The cells were lysed with RIPA buffer (Cell signaling technology, #9806) with proteinase inhibitors (GenDEPOT, #P3100-001) and phosphatase inhibitors (GenDEPOT, #P3200-001). An equal amount of total protein was denatured in electrophoresis loading buffer for analysis via sodium dodecyl sulfate-polyacrylamide gel electrophoresis (SDS-PAGE). The separated proteins were transferred from the gel onto polyvinylidene difluoride (PVDF) membranes (0.45μm, Merck Millipore, #IPVH00010) and incubated with primary antibodies overnight at 4°C. They were incubated with an appropriate horseradish peroxidase–conjugated secondary antibody (Bethyl #A90-116P, #A120-101P) to visualize the proteins using ECL reagents (Thermo Fisher, #1859698, #1859701). Detailed information of antibodies is listed below

- Anti-FBLN5: Abcam, ab66339
- anti-E-Cadherin: Cell Signaling #3195
- anti-N-Cadherin:Cell Signaling #13116
- anti-Vimentin: Cell Signaling #5741
- anti-Snail: Cell Signaling #3879
- anti-Slug: Cell Signaling, #9585
- anti-FAP: Cell Signaling #66562
- anti-pCREB: S133; Cell Signaling #9198
- anti-CREB: Cell Signaling #9197
- anti-pGSK3α β: S21/S9; Cell Signaling, #8566
- anti-GSK3αβ: Cell Signaling, #5676
- anti-GAPDH: Santa Cruz, sc47724

Immunofluorescence

Antibodies for fibulin-5 (Abcam #ab66339), Vimentin (Cell Signaling #5741), FAP (Cell Signaling #66562), PDGFα (Cell Signaling #3174), PDGFβ (Cell Signaling #3169), S100A4 (Cell Signaling #13018), α-smooth muscle actin (Cell Signaling #19245) and DAPI (Vector #H-1200) were used for immunofluorescence staining according to the method previously described [4].

siRNA construction and transfection

All siRNA duplexes were obtained from Bioneer (Daejeon, Republic of Korea). Cells were cultured in serum-free medium at 37°C until they reached approximately 70% confluence. The following day, cells were transfected with either a negative control siRNA (SN-1012) or si*FBLN5* using TransIT-X2 Dynamic Delivery System (Mirus Bio LLC). The sequences for the *FBLN5*-targeting siRNAs were synthesized as follows: Sense: 5' GGCAGAGAAUUUUACAUGCGGCAAAAG-3' and Antisense: 5'-UUUGCCGCAUGUAAAAUUCUCUGCCAU-3'.

Chemicals and proteins

Compound 3i (666-15; Sigma-Aldrich #5383410001) and KG501 (Selleck chemicals LLC #S8409) were used as a CREB inhibitors. Recombinant Human Fibulin-5 Protein (R&D system # 9006-FB-050) was used to simulate secreted fibulin-5 in the culture media.

Invasion and Migration assay

Trans-filter migration and invasion assays were performed as previously described [5].

Human phospho-kinase array

Protein phosphorylation was quantified using the Proteome Profiler Human Phospho-Kinase array kit (R&D Systems, ARY022B) according to the manufacturer's instructions. The signals were detected using ECL reagents (Thermo Fisher #1859698, #1859701), and the resulting images were quantified by densitometry using ImageJ software to measure phospho-protein levels.

Wound scratch assay

GFP-expressing MKN-45 stable cells were seeded in a 96-well microplate for both mono-culture and co-culture groups within the live cell imaging system. In the co-culture group, MKN-45 cells were mixed either with CAFs or NAFs at a 5:1 ratio. Scratch wounds were created using the WoundMaker™ (Essen Bioscience) placed over the microplate.

Enzyme-linked immunosorbent assay (ELISA)

Process of conducting ELISA on plasma samples from those undergoing gastric resection surgery or endoscopic treatment was approved by the SNUH institutional review board (IRB #2006-060-1112). The patient cohort included individuals with DGC as well as non-cancer patients with gastric or duodenal polyps. Plasma samples were collected preoperatively from patients and ELISA was performed using Fibulin-5 ELISA kit (antibodies-online, ABIN6955903) according to the manufacturer's instructions.

1 Perfetto, L. *et al.* SIGNOR: a database of causal relationships between biological entities. *Nucleic Acids Res* **44**, D548-554 (2016).

2 UKCCCR guidelines for the use of cell lines in cancer research. *Br J Cancer* **82**, 1495-1509 (2000).

3 Kang, S. *et al.* Tumorigenic mechanisms of estrogen and Helicobacter pylori cytotoxin-associated gene A in estrogen receptor α-positive diffuse-type gastric adenocarcinoma. *Gastric Cancer* **25**, 678-696 (2022).

4 Choi, S. I. *et al.* RETRACTED: CDX1 Expression Induced by CagA-Expressing Helicobacter pylori Promotes Gastric Tumorigenesis. *Mol Cancer Res* **17**, 2169-2183 (2019).

5 Shin, J.-Y. et al. MicroRNA 135a Suppresses Lymph Node Metastasis through Down-Regulation of ROCK1 in Early Gastric Cancer. PLOS ONE 9, e85205 (2014).
